# Behavioral responses to cow and calf separation: separation at 1 and 100 days after birth

**DOI:** 10.5713/ab.22.0257

**Published:** 2022-11-14

**Authors:** Sarah E. Mac, Sabrina Lomax, Cameron E. F. Clark

**Affiliations:** 1 Livestock Production and Welfare Group, School of Life and Environmental Sciences, Faculty of Science, The University of Sydney, Camden, NSW 2570, Australia

**Keywords:** Cattle-maternal-filial Bond, Cow-calf rearing, Maternal Separation, Vocalization, Weaning

## Abstract

**Objective:**

The aim was to compare the behavioral response to full separation of cows and calves maintained together for 100 days or 24 h.

**Methods:**

Twelve Holstein-Friesian cow-calf pairs were enrolled into either treatment or industry groups (n = 6 cow-calf pairs/group). The treatment cows and calves were maintained on pasture together for 106±8.6 d and temporarily separated twice a day for milking. The Industry cows and their calves, were separated within 24 h postpartum. Triaxial accelerometer neck-mounted sensors were fitted to cows 3 weeks before separation to measure hourly rumination and activity. Before separation, cow and calf behavior was observed by scan sampling for 15 min. During the separation process, frequency of vocalizations and turn arounds were recorded. At separation, cows were moved to an observation pen where behavior was recorded for 3 d. A CCTV camera was used to record video footage of cows within the observation pens and behavior was documented from the videos in 15 min intervals across the 3 d.

**Results:**

Before separation, industry calves were more likely to be near their mother than Treatment calves. During the separation process, vocalization and turn around behavior was similar between groups. After full separation, treatment cows vocalized three times more than industry cows. However, the frequency of time spent close to barrier, standing, lying, walking, and eating were similar between industry and treatment cows. Treatment cows had greater rumination duration, and were more active, than industry cows.

**Conclusion:**

These findings suggest a similar behavioral response to full calf separation and greater occurrence of vocalizations, from cows maintained in a long-term, pasture-based, cow-calf rearing system when compared to cows separated within 24 h. However, further work is required to assess the impact of full separation on calf behavior.

## INTRODUCTION

In dairy systems, calves are commonly removed within 24 h postpartum and subsequently hand reared either in housing or pasture systems [1] to increase saleable milk, decrease disease transfer, increase ease of management around milking [2,3], and increase calf monitoring [2]. These calves can either be housed individually or in groups of two or more and fed milk using artificial teats or buckets, however these rearing systems are perceived poorly by the public stating it is unnatural, causes emotional stress and poor health [4–6]. An alternative to such systems is the maintenance of the calf with the cow, but existing research has predominantly focused on housed systems [7–9] with either full [7,8,10] or part-time contact [3,11]. In this regard, there is a paucity of data pertaining to the maintenance of cows and calves together on pasture until weaning.

Cow-calf contact systems allow calves to access relatively high volumes of milk, and calves maintained with the cow typically show greater rates of body weight gain when compared to rearing calves on artificial teat buckets [2,12] but the impact on cow milk production varies [2]. Greater calf milk consumption can increase milk production in the first lactation [13] but maintaining the cow and calf together has inconclusive health impacts compared to early separation [14]. Longer duration cow-calf systems can be associated with increased labor requirements for physical separation of cows and calves for milking, decreased calf habituation to humans, and increased stress around full separation [3,12,15].

Greater stress response of cows and calves to full separation has been reported when maintained together for 4 [10], 14 [7], and 63 [11] days as compared to the industry common practice of separation within 24 h [7,10,11]. Beef cows and calves separated between 6 and 8 months postpartum have been shown to exhibit stress responses to weaning separation including vocalization [16–18], longer standing [17] and walking times [16,17], and decreased rumination [16–18]. The maternal bond that is formed within the first 5 min, increasing with suckling and social interactions [19] but data on cow and calf behavior response to full separation after more than 2 weeks is lacking.

Research investigating the effects of long-term temporary separation for milking on the impact of dairy cow behavioural stress response at full separation may provide insight into the implementation of pasture-based cow-calf systems. Our objective was therefore to compare the behavioral response to full separation between cows that were maintained with their calf for 100 days and cows separated from their calf within 24 h.

## MATERIALS AND METHODS

This experiment was conducted at The University of Sydney’s commercial dairy farm between July and August 2019 in accordance with the University of Sydney Animal Ethics Committee regulations (Protocol 2018/1462).

### Animal management and monitoring

Twelve Holstein-Friesian cow calf pairs were used in this study and allocated to one of two groups (treatment, n = 6 pairs; industry, n = 6 pairs). Treatment cow-calf pair management during the first 100 days of lactation are described by Mac et al [12]. Briefly, treatment cows (3.5±1.4 lactation) and calves were maintained together on pasture for over 100 d [12]. In this system cows were temporarily separated twice a day for milking while their calves remaining in the paddock. Temporary electric tape fences were used for strip grazing with fresh pasture allocated daily and cows restricted one section of the paddock. Treatment calves had access to the entire paddock by walking under the electric tape. At the time of enrollment, treatment calves weighted 206±13.9 kg with an average daily gain of 1.4±0.2 kg/d and treatment cows were 106±8.6 days in milk producing an average of 11.2±5.6 kg/cow/d when maintained with their calves and 31.3±8.6 kg/d after separation [12]. Industry cows (3.7±1.8 lactation) representing the industry standard (calf removed within 24 h postpartum; [9,11]), were selected from the main herd and separated from their calf in the calving paddock within 24 h of calving. Two dry cows were introduced into the treatment paddock and the calving paddock (n = 2 per group) a minimum of 2 weeks before cow-calf separation for habituation to act as companion cows during post-separation observations allowing observed cows to display natural herd socialization behavior [20].

All cows were fitted with a collar-based triaxial neck accelerometer (SCR HR-LDn, Netanya, Israel) 21 days before separation. The SCR neck collar measured rumination and general activity with data output provided as summed 2-h intervals (Heatime Pro+; SCR HR-LDn, Israel) validated by Schirmann et al [21]. For each observation period post separation, cows were moved to 20 m×20 m observation pens to monitor their behavioral response for three days. Each pen had a focal cow (alternating between treatment and industry cows) along with one companion dry cow that was not observed. Pens were located 1 km apart from each other to prevent behavioral impact between observation cows. On the day of full separation, two treatment cows (separated from their calves at 106±8.6 days old) or two industry cows (separated within 24 h postpartum) were moved into separate observation pens and observed for 72 h using CCTV cameras (as described below) before being returned to the main milking herd. The behavior of the dry cows was not recorded. Each pen consisted of 1 observed cow (treatment or industry), 1 dry cow, straw bedding, a water trough and hay feeder where cows were provided *ad libitem* lucerne hay. Treatment calves (males) were immediately transported to a local abattoir where they were processed within 24 h. Industry calves (male and females) were separated and reared on farm in a calf shed preventing visual or auditory contact with the cows. During observation cows were milked twice a day at 0400 h and 1230 h in a rotary, robotic milker (AMR mk1; DeLaval, Botkyrka, Sweden), while the dry cows remained in the observation pens.

### Behavioral observations

Behavior was recorded in three periods i) Pre-separation: 15 min before full separation of cow and calf; ii) During separation: from the time the cows left the yards to the time the cows entered their respective pens (<5 min); and iii) Post separation: interval recording for 72 h following full separation recorded through video. Pre-separation cow and calf behavior was recorded in their designated paddocks (treatment, treatment paddock; industry, calving paddock). Behavior pre-separation and during separation (Table 1) was observed using a 0/1 binomial scan sampling every min for 15 min. Directly after pre-separation observations, cows and their calves were moved into the yards of the treatment paddock. Calves were held in the yards as the cows were drafted out and walked to their designated observation pens. During this process, continuous observations were conducted to record cow vocalizations and attempts to reunite with their calves (Table 1) until cows entered their respective observation pens. In the observation pens, cow behavior was continuously recorded using CCTV cameras (NVW-490; Swann Security, Melbourne, Australia) with cameras located at the pen entrance. Video data were analyzed in 15 min segments at 0 (cow first entering the observation pen), 1, 3, and every 3 h thereafter, for 72 h post-separation and scored using open-source software (BORIS Behavioral Observation Research Interactive Software, Life Sciences and Systems Biology, Via dell’Accademia Albertina, Edition 7.8, Torino, Italy). Cows were observed for proportion of time spent eating, close to barrier (≤2 m from paddock entrance), walking, lying and standing (Table 2). Vocalization behavior was measured as total frequency within each timepoint.

### Statistical analysis

Sensor and behavioral observation data occurring in the pre- and during separation time periods were analyzed using a generalized linear mixed model (GLMM) in GenStat 16th edition (VSN International, Hemel Hempstead, UK). Cow sensor behavior data were summed per day and analyzed by groups across the 3 d before separation (pre-calf removal), the 3 d directly after separation (calf removal) and the 3 d after returning to the main milking herd (return to herd) with day 0 representing day of separation. Cow number/calf number was the random effect for each respective behavioral data analysis. Behavior recorded in pre-separation and during separation observation periods were analyzed for total occurrence by group. CCTV behavior data was exported as time budgets, giving frequency and duration of each behavior in each 15 min interval. CCTV behavioral data and sensor data were analyzed using a restricted maximum likelihood model in GenStat 16th edition (VSN International, UK). The treatment units of the behavioral data for CCTV were time period, behavior frequency and behavior duration and analyzed by group in each timepoint segment and summed across days.

## RESULTS

### Pre-separation behavior

Cow behavior observed during pre-separation and during separation time periods are presented as mean probability in Table 3. During the pre-separation observations, industry cows were 9 times more likely to be observed in close proximity (≤2 m) to their calf than treatment cows. Industry cows were observed standing the entire observation period as compared to a third of the time for treatment cows. Only two suckling events were recorded, both by the same industry calf. No difference was observed in turn around and vocalization behaviors during the physical separation.

### After separation behavior

After separation eating, lying, walking, and standing behavior were similar for treatment and industry cows. Cows spent more time standing on day 1 as compared to days 2 and 3 (p<0.05). At time point 0 (the 15 min directly after entering the observation pen), treatment cows had greater eating durations than industry cows and were observed eating for 8 times more during that proportion of time (p<0.001) with a mean percentage of occurrence 81% and 10%, respectively. Cows were observed for the greatest period of time close to the barrier at 18 and 27 h post separation (p<0.001). Treatment cows vocalized three times more than industry cows across the 72 h of observation (p<0.001).

### Neck tag sensor data

Rumination and activity of treatment and industry cows before and after calf removal and when returned to herd is represented in Table 4. Treatment cows spent more time ruminating than industry cows after separation except for the day directly following separation (p<0.05; day 1, Table 4). Treatment cow rumination time was consistent across all time periods. Industry cows spent less time ruminating after calf removal and when returned to the herd than before calf removal by 117.2 (±30.8) and 100.1 (±41.9) min, respectively (p<0.05). Treatment cows were more active than industry cows during the calf removal time period but had similar activity times during pre-calf removal and after they were returned to the herd. Treatment and industry cows had similar activity levels on the day of separation (day 0, Table 4) but treatment cows were more active on day 1 and 2 compared to industry cows. The 2 days after calf removal, treatment cows were more active compared to pre-calf removal and returning to the herd (p<0.05; day 1 and 2, Table 4). Industry cow activity was consistent across all time periods.

## DISCUSSION

Cow behavioral response was similar across groups during the separation process and the 3 d after separation despite the longer cow-calf contact of the treatment cows. The age of calves impacted cow behavioral response throughout the entire process of cow-calf separation. Treatment cow vocalization, rumination and activity were more than industry cows with all other behaviors similar between the two groups resulting in evaluations of the data inconclusive.

Before separation, treatment cows were near their calves for less of the observed time as compared to industry cows. Similarly, the proximity of beef cows to their calves was shown to increase from an average of 7 to 38 meters over 125 days from birth [22]. Greater distance represents the increasing social and nutritional independence of our calves with age/at that age/100 d. A reduction in suckling events and increased grazing behavior was previously reported for these treatment calves during the first 100 days of life [12] which aligns with the greater distances from their mother observed in the current study. Treatment cows were observed standing less than industry cows before separation which is also likely due to the age of treatment calves as they were observed with lesser standing times and no suckling events pre-separation. Industry calves were nutritionally dependent on their mother but the nutritional dependance on their mothers decreases as calves age resulting in a reduction in the maternal-filial bond [23]. Our results suggest longer cow-calf contact may provide an advantage over current contact periods of cow-calf systems due to an increase in calf independence.

During physical separation of cows from calves, the total number of turn arounds were similar between treatment and industry cows. Previous research documenting cow response during the physical separation process is lacking. It was hypothesized that the treatment cows would have a greater distress response based on previous work [7,10,11], however, despite the longer duration of calf contact, our findings contrast preceding reports. An explanation for this contrast could be due to a more nutritionally independent calves have a reduced stress response to cow and calf separation [24]. Both groups of cows had high motivation to return to their calves indicated by vocalizations and turn arounds. This motivation to return to their calf for industry cows could be attributed to the accumulation of stress from calving [25,26] relocation [27,28], and the stress of early calf separation [10,18,29]. The response of treatment cows to physical separation from their calves was unexpected as they were habituated to being separated twice daily for routine milking [12]. Treatment cows had habituated to the routine of temporary separation twice a day [12] for over 100 d and calves were more independent as reported in pre-separation observations and documented in Mac et al [12]. However, the increased vocalization/turnarounds could be associated with the change in routine in addition to the change in the direction they were moved (away from, instead of toward the dairy) which may have contributed to stress. As calves were processed immediately for meat quality and rumen development [12], further work is needed to determine the stress responses of calves to full separation at weaning following extended cow-calf contact in dairy systems.

Treatment cows vocalized more than industry cows during the 72 h following full separation. It should be noted, previous work evaluating animal behavior in cow-calf systems is limited, with studies evaluating cows and calves for a maximum duration from 4 to 63 d as compared to 100 days in the current work. Similarly, cows separated from their calves at 4 [10] and 14 [7] days displayed greater frequency of vocalizations after separation as compared to groups separated at 1 day and 6 h. Vocalization has been linked to physiological [30] and behavioral [31] signs of stress, and associated with distress following cow-calf separation [10,18,29]. The maternal-filial bond forms within the first 5 min [19] and increases with physical contact, suckling and grooming behavior [3, 32]. Previous research suggests that within the first 21 h [10] and 51 h [18] of separation, the greatest number of vocalizations occur approximately 9 h post separation. However, the number of vocalizations in the current work were consistent across days and time periods. Conversely, beef cows maintained with their calves for 45 days postpartum were reported to vocalize less 72 h after calf separation when compared to cows separated at 25 days [29] and was accredited to maternal needs of the younger calves. Without other measurements, the motivation behind the vocalizations cannot be concluded as they could be stressed from other factors other than calf separation.

There was no difference in eating, close to barrier, standing, walking or lying behaviors between the groups of cows. Previous literature evaluating cow-calf separation reported longer standing times and greater duration of time spent with their head out of the pen for cows maintained with their mothers for 4 [10] and 14 days [7], as compared to cows and calves separated at 6 and 24 hours postpartum. The longevity of time the treatment cows were maintained with their calves could account for these differences. In beef systems, cows with a greater number of nursing bouts before weaning at 6 months exhibited a greater distress response to separation as observed by an increase in pacing and decrease in eating behaviors [18,33]. Treatment calves showed decreased suckling bouts leading up to full separation [12] and the increase in calf independence could explain the absent/reduced response of treatment cows to full separation. Similarly, Grøndahl et al [8] anecdotally reported vocalizations to be the main indicator of separation distress for cows that had been maintained with their calves for 6 to 8 weeks.

Treatment cows spent a greater time ruminating as determined by sensors compared to industry cows for the 3 d after calf removal. It is important to note that treatment cows and industry cows were at different stages of lactation, which likely contributed to divergent behavioral responses. Rumination decreases around calving [21,34,35] due to decreased feed intake [21,36] and can explain the lower rumination time of the industry cows on day of separation when compared to treatment cows. However, Adin et al [34] and Soriani et al [35] reported a steady increase in rumination time the days following calving which was not observed in this study. Rumination is a positive welfare indicator [37,38], and decreases during stressful events [39,40], therefore the increase observed in treatment cows suggests a lower impact of full separation. Previous research has shown associations between milk yield and rumination, lying, standing, and eating times [41,42], but not vocalizations. Although, these production behaviors are likely being confounded by stage of lactation, dependency of cows’ calves and sample size, our results do provide new insights into the impact of a long-term, cow-calf, pasture-based system.

Treatment cows had greater sensor derived activity data across the 3 d following calf removal. Although an increase in activity has been linked to greater levels of stress, [16,43,44], the valence of this behavior cannot be assumed without context of whether it is indicative of negative affective state. Sensor derived behaviors rely on changes in the axis orientation of the device, which can provide detail on frequency of postural changes (interpreted as activity) but does not provide information on the type of behavior being performed. The sensor derived increase in activity did not align with the visually observed behaviors as the complete time budget of the cows was not represented which may be due to the sample size, frequency of sampling and the limited number of behaviors included in the ethogram. Behaviors included in the ethogram were specifically linked to stress, allowing the behaviors recorded by the accelerometer to include behaviors not recorded in the ethogram such as placid behaviors. Increased activity can be related to both positive and negative welfare states such as allogrooming and play behavior. An increase in visually observed activity has been reported in cows separated from their calf at 14 days postpartum as compared to those separated at 1 day [7] although both of these timepoints are considerably shorter than the 100 days of the current work. Further work is required on a larger sample size to evaluate the long-term impact of abrupt separation on cows for the current systems, and to determine the impact on calves.

## CONCLUSION

We investigated the impact of a novel pasture-based cow-calf rearing system on cow behavior at full separation when maintained with their calf for greater than 100 d compared to cows separated within 24 h representing common industry practice. The duration of time spent with calf did not appear to impact cow stress response during the physical separation process. Past literature suggests treatment cows would have a greater behavioral stress response to full separation when compared to industry cows, however, we observed similar behavioral response (except for vocalizations). This phenomenon could be attributed to the increase in independence of treatment calves. After full separation, treatment cows were observed ruminating more suggesting a lower distress behavior when compared to industry cows. However, treatment cows were more active and vocalized more than industry cows potentially suggesting greater stress response, but the valence of these behaviors cannot be determined. Through the results of this study, cow behavior linked to production (eating, lying, and rumination) was not negatively impacted due to length of time with their calf. Further research on larger sample sizes and observing calf response is necessary to illustrate the complete impact of long-term, pasture-based cow-calf systems on full separation.

## Figures and Tables

**Table 1:** Ethogram with the description of cow and calf behaviors measured during live observations categorized by observation period and modified from

Behavior	Definition	Observation periods
Close to calf	Cow positioned within one cow body length (2 m) of her own calf	Pre-milking, post milking
Nursing	Cow’s calf has his nose or mouth in contact with mother’s udder followed by sucking on a teat with mouth	Pre-milking, post milking
Turn arounds	Cow turns her head, neck, and the front of her chest oriented toward calf location	Temporary separation process, Parlor herding
Vocalization	Audible sound coming from animal’s mouth	Pre-milking, temporary separation process, parlor herding, temporary separation, post milking
Standing	Animal’s torso is not in contact with ground, all weight supported by hooves (includes standing still and moving (e.g. walking, running) while in a non-recumbent position	Pre-milking, temporary separation, post milking
Grazing	While standing, the animal has its head angled down (below withers) and moving muzzle (nose and mouth) along close to grass (within 10 cm) and taking grass into the mouth, followed by chewing	Pre-milking, temporary separation, post milking
Suckling	Calf having nose or mouth in contact with an unrelated cow’s udder followed by sucking on a teat with mouth	Pre-milking, post milking

Modified from Flower and Weary [7] and Weary and Chua [10].

**Table 2:** Ethogram with the descriptions of cow behaviors measured using CCTV footage during the 3-day observations after full separation

Behavior	Definition
Eating	While standing, taking hay into the mouth followed by moving jaw in a chewing motion and swallowing
Vocalization	Audible sound coming from animal’s mouth
Close to Barrier	Positioned so that any part of the body is within 2 m from the entrance of the pen
Walking	All four hooves of the cow must move once without pause moving away from current location
Lying	In recumbent position, not standing on hooves to support weight
Standing	Animal’s torso is not in contact with ground, all weight supported by hooves (includes standing still and moving (e.g. walking, running) while in a non-recumbent position

Modified from Flower and Weary [7] and Weary and Chua [10].

**Table 3:** Probability of behaviors during pre-separation and during separation for treatment and industry cows

Group	Observation period			

	Pre-separation*		During separation	
	
	Close	Standing	Vocalization	Turn arounds
Treatment	12.2^a^	33.7^a^	7.9^a^	2^a^
Industry	91.3^b^	99.6^b^	6.6^a^	4^a^

a,b Means within a column with different subscripts differ (p<0.05)*

**Table 4:** Mean daily cow rumination and activity behavior, collected using neck accelerometers, categorized by treatment for time periods: before, during and after full separation

Items	Days	Rumination (min/d)		Activity (min/d)	
	
		Treatment	Industry	Treatment	Industry
Pre-calf removal	−3	420^a^	575^b^	488^a^	459^a^
	−2	468^a^	437^a^	521^a^	561^a^
	−1	469^a^	450^a^	560^a^	598^a^
Calf removal^1)^	0	530^a^	344^b^	697^a^	632^a^
	1	499^a^	404^a^	736^a^	534^b^
	2	497^a^	361^b^	657^a^	467^b^
Return to herd	3	498^a^	344^b^	584^a^	534^a^
	4	498^a^	388^b^	552^a^	496^a^
	5	545^a^	428^b^	511^a^	524^a^

1) Day of separation = 0.

a,b Means within a row with different subscripts differ (p<0.001).
